# Editorial: Methodological issues in consciousness research

**DOI:** 10.3389/fpsyg.2023.1217732

**Published:** 2023-06-06

**Authors:** Luca Simione, Antonino Raffone, Roumen Kirov, Morten Overgaard, Aviva Berkovich-Ohana, Axel Cleeremans

**Affiliations:** ^1^Institute of Cognitive Sciences and Technologies, CNR, Rome, Italy; ^2^Faculty of Interpreting and Translation, Università degli Studi Internazionali, UNINT, Rome, Italy; ^3^Department of Psychology, Sapienza University of Rome, Rome, Italy; ^4^Institute of Neurobiology, Bulgarian Academy of Sciences (BAS), Sofia, Bulgaria; ^5^CFIN, Department of Clinical Medicine, Aarhus University, Aarhus, Denmark; ^6^Faculty of Education, University of Haifa, and Brain and Behavior Hub, Haifa, Israel; ^7^Center for Research in Cognition and Neuroscience (CRCN), ULB Neuroscience Institute (UNI), Université libre de Bruxelles, Brussels, Belgium

**Keywords:** methodologic challenge, self-control, online research, EEG, perceptual awareness negativity, self-awareness

The study of consciousness has been greatly shaped by the development of increasingly compelling methods to measure it, from Wundt (1897) introspection at Lipsia (Wundt, 1897), to the studies of brain lesions of the middle of the past century (see LeDoux et al., 2020) and, finally, to the search for the neural correlates of consciousness through EEG and fMRI studies (Koch et al., 2016). As methods have had such a prominent role in guiding and orienting the research on consciousness, we felt a Research Topic on Methodological Issues in Consciousness Research was timely and relevant. We aimed to assemble different contributions to the field, spanning different experimental methods, from the well-established EEG paradigm to the new zest for online behavioral studies. In the following, we offer a brief overview of the contents of each contribution.

The commentary by Moayery points out three critical issues with the model of self-control in consumer behavior as proposed by Vosgerau et al. (2020), in which self-control failures derive from the choice of violating superordinate long-term goals, putting a great emphasis on overt or conscious processes. While recognizing the value of this position, Moayery's article originally underlines the following three critical points in the formulation of Vosgerau et al. (2020), emphasizing the role of unconscious processes in determining consumer behaviors. First, the lack of self-control does not always derive from a deliberate choice between alternative goals, as people often fail to self-monitor their actions or intentions. Second, while ego-depletion theory suffers from several shortcomings, it addresses a number of real-life phenomena that should be taken into account and not rejected. Third, Vosgerau et al. (2020) model ignores the contribution of impulsive mechanisms in determining human behavior. Overall, this commentary highlights the need to consider a more balanced perspective on the role of conscious and unconscious determinants of self-control in future research.

A large number of studies conducted in the last years have been exploiting the possibility of using online research tools instead of laboratory ones, as they guarantee easier and faster access to large groups of participants. However, the validity of such online instruments in consciousness studies is still a matter of scientific debate. In this framework, the article by Hirao et al. explores the reliability of online experiments for collecting data about perception and impressions of faces. To this aim, they conduct an experimental study with both a typical laboratory sample and two online samples. They compare the samples' responses regarding three visual perceptual features and 16 items regarding impressions of the face, such as trustworthiness, honesty, and attractiveness. Overall, they find a moderate to high correlation between the scores assessed in the online and in the in-person samples and a very limited rate of mismatch between the samples. However, they find that the differences in the average scores of the stimuli were smaller in the online assessment than in the laboratory one, suggesting the need for a larger sample while conducting online research. This article, then, is valuable in providing practical guidelines for future online studies investigating the perception and impression of faces.

Consciousness research makes large use of neuroimaging methods, as they can reveal how the brain works in different consciousness states and for elaborating different contents. This Research Topic includes three articles exploring the limits of the actual neuroscientific methods in consciousness studies and proposing new frameworks for their improved use. The first article by Koculak and Wierzchon presents a critical re-thinking about the use of the so-called “resting-state” paradigm, in which participants are scanned with an fMRI while not receiving any external stimulation and having no straightforward task. They argue that this paradigm, while extremely useful in the context of clinical application for the diagnostics of various disorders of consciousness, could be made more useful for a non-clinical scientific approach. In particular, they propose adding a type of experience sampling during the paradigm to connect neural activity and mental contents, and to mix the resting-state paradigm with tasks, as this would further increase the ecological validity of this assessment.

Investigations about the neural correlate of consciousness also employ the event-related potential (ERP) technique. Recently, it has been proposed that an early negative ERP component may reflect the awareness of stimuli, in terms of perceptual awareness negativity (PAN; Dembski et al., 2021). PAN has been extensively investigated by Koivisto and colleagues (Koivisto and Revonsuo, 2007, 2010; Koivisto and Grassini, 2016). Bola and Doradzinska critically review the evidence in favor of PAN as a marker of consciousness. In particular, as many contradicting results point out the possibility that PAN mostly reflects attentional processes rather than awareness, they question whether the “A in PAN indeed stands for awareness and not attention” (Bola and Doradzinska; p. 3). PAN shares several features with a typical attentional component such as the N2 posterior-contralateral (N2pc). Thus, the authors suggest investigating PAN using a more falsification-oriented approach, thus effectively dissociating attention from awareness. As this literature is in its infancy, we hope that this opinion article could inspire a new line of investigations in the search for the neural correlate of consciousness.

In their article, Paoletti et al. address the interesting problem of the electroencephalographic correlates of different levels of self-awareness. Starting from the definition of different levels of the self as represented as concentric circles, from a peripheral narrative self to a central state of overcoming the self (a third state proposed by the authors and characterized by a complete absence of any sense of self), Paoletti et al. propose a new hypothesis. They argue that “moving” toward the center of the model is related to a gradual slowing of the EEG frequencies associated with each state of the self. To support their hypothesis, they further review a number of EEG studies on different meditative states and their associated levels of self-awareness. From this mini-review, they report evidence consistent with their hypotheses, with slower EEG frequencies detected during non-dual (emptiness) meditation, a meditative practice aimed at experiencing a form of pure awareness or absence of the self, resembling the overcoming of the self state proposed by the authors. However, further studies should be conducted to test this hypothesis.

## Conclusion

In conclusion, this collection addresses many fundamental issues that have emerged over the last few years in the quest to understand consciousness, including problems related to self-awareness and the implications of recent neuropsychological studies. Even if these articles open up more scientific questions than they answer, they can contribute to the debate around the methodological foundation of consciousness research. The epistemology of consciousness studies is still in search of clear definitions and methods (Harman, 1994), to which we hope to contribute with this Research Topic.

## Author contributions

All authors listed have made a substantial, direct, and intellectual contribution to the work and approved it for publication.
